# Meteorological Report

**Published:** 1881-08

**Authors:** 


### METEOROLOGICAL REPORT FOR JUNE, 1881.

| RAIN. DATE | RAIN. IN. | Mean daily Barometer. | Mean daily Thermometer | Mean daily Humidity. | Maximum Thermometer | Minimum Thermometer | Prevailing direction of Wind. | Maximum hourly velocity of Wind, (Daily.) |
|---|---|---|---|---|---|---|---|---|
| 1 | .12 | 29.828 | 75.3 | 64 7 | 85.0 | 66.8 | S. W. | ......... |
| 2 | .85 | 29.799 | 73.2 | 77.3 | 80.0 | 67.0 | N. W. | ......... |
| 3 | 0 | 29.809 | 76.1 | 53.7 | 83.2 | 66.8 | W. | ......... |
| 4 | 0 | 29 958 | 65.6 | 56.3 | 74.0 | 61.0 | N. W. | ......... |
| 5 | 0 | 29.957 | 72.2 | 51.0 | 81 0 | 58.5 | W. | ......... |
| 6 | 0 | 23.994 | 77.2 | 51 0 | 85.2 | 66,0 | W. | ......... |
| 7 | 0 | 30 042 | 78.2 | 58.3 | 88.8 | 69.0 | W. | ......... |
| 8 | .52 | 29.969 | 71.0 | 86.7 | 77.2 | 69.2 | S. W. | ......... |
| 9 | .35 | 29.911 | 74.6 | 70.0 | 81.8 | 63.2 | N. W. | ......... |
| 10 | 0 | 29.955 | 74.9 | 56.7 | 81.5 | 67.2 | N. W. | ......... |
| 11 | 0 | 30.029 | 73.4 | 57.7 | 81.5 | 63.0 | N. E. | ......... |
| 12 | 0 | 30.079 | 74 7 | 70 0 | 81 5 | 65,0 | E. | ......... |
| 13 | 0 | 30.062 | 81.7 | 56.0 | 87 0 | 71.0 | E. | ......... |
| 14 | ...... | 30.078 | 79.5 | 63.3 | 87.8 | 74.0 | N. W. | ......... |
| 15 | 0 | 30.111 | 80 9 | 55.9 | 89.8 | 69,2 | N. | ......... |
| 16 | 0 | 30.090 | 83.3 | 48.7 | 90.0 | 71.2 | N. E. | ......... |
| 17 | 0 | 30.005 | 87.1 | 39.0 | 92.5 | 75.2 | N. | ......... |
| 18 | 0 | 29.967 | 85.9 | 49.0 | 94.8 | 78.0 | N. W. | ......... |
| 19 | 0 | 29.963 | 85.3 | 51.3 | 91.8 | 73.0 | N. W. | ......... |
| 20 | 0 | 29.927 | 87.1 | 47 0 | 93.8 | 73.8 | N. W. | ......... |
| 21 | 0 | 29.827 | 83.0 | 53.7 | 92.8 | 75.0 | N. W. | ......... |
| 22 | 0 | 29.827 | 77.4 | 63 0 | 85.2 | 69.8 | N. W. | ......... |
| 23 | 0 | 29.984 | 75 2 | 58.3 | 83.0 | 64.5 | N. E. | ......... |
| 24 | ...... | 30.052 | 77.1 | 63.7 | 89.5 | 70.8 | S. | ......... |
| 25 | .04 | 30,036 | 77.5 | 78.3 | 81.8 | 70.2 | E. | ......... |
| 26 | .01 | 30.057 | 77.4 | 78.0 | 83.0 | 71 2 | N. W. | ......... |
| 27 | .55 | 30.074 | 77.6 | 68.0 | 90.2 | 71.0 | W. | ......... |
| 28 | .02 | 30.028 | 82.2 | 68.3 | 86.2 | 69 0 | W. | ......... |
| 29 | 0 | 29 982 | 84.0 | 59.7 | 91.5 | 74.2 | N. W. | ......... |
| 30 | 0 | 29 950 | 86.7 | 55.3 | 91.8 | 76,8 | N. W. | ......... |
| ... | ...... | ......... | ...... | ...... | ...... | ...... | ......... | ......... |
| Sums | | 29.978 | 78.5 | 60.3 | 86.1 | 69.4 | N. W. | ......... |

Highest Barometer, 30.149, 16th.
Lowest Barometer, 29.690, 2d.
Monthly range of Barometer, 0.459.
Highest Temperature, 94 8°, 18th.
Lowest Temperature, 58.5°, 5th.
Monthly range of Temperature, 36.3°.
Greatest daily range of Temperature, 22:5°, 5th.
Lowest daily range of Temperature. 8.0°, 8th.
Mean of Maximum Temperature, 86,1°.
Mean of Minimum Temperatnre, 69.4°.
Mean daily range of Temperature, 16.7°.
Total rainfall or melted snow, 2.46 inches.
Prevailing wind, Northwest.
Total movement of wind, 5,331 miles.
Maximum velocity of wind and direction, 35 miles per hour, N. W., 27th.
Number of cloudy days on which rain fell, 3.
Number of cloudy days on which no rain fell, 2.
Total number of days on which rain or snow fell, 9.

